# Normal and dysregulated crosstalk between iron metabolism and erythropoiesis

**DOI:** 10.7554/eLife.90189

**Published:** 2023-08-14

**Authors:** Yelena Ginzburg, Xiuli An, Stefano Rivella, Adam Goldfarb

**Affiliations:** 1 Division of Hematology and Medical Oncology, The Tisch Cancer Institute, Icahn School of Medicine at Mount Sinai New York United States; 2 LFKRI, New York Blood Center New York United States; 3 Department of Pediatrics, Division of Hematology, The Children’s Hospital of Philadelphia Philadelphia United States; 4 Cell and Molecular Biology affinity group (CAMB), University of Pennsylvania Philadelphia United States; 5 Raymond G. Perelman Center for Cellular and Molecular Therapeutics at the Children’s Hospital of Philadelphia Philadelphia United States; 6 Penn Center for Musculoskeletal Disorders at the Children’s Hospital of Philadelphia Philadelphia United States; 7 Perelman School of Medicine, University of Pennsylvania Philadelphia United States; 8 Institute for Regenerative Medicine at University of Pennsylvania Philadelphia United States; 9 RNA Institute at University of Pennsylvania Philadelphia United States; 10 Department of Pathology, University of Virginia Charlottesville United States; Augusta University United States; Augusta University United States

**Keywords:** erythropoiesis, iron deficiency, anemia, polycythemia

## Abstract

Erythroblasts possess unique characteristics as they undergo differentiation from hematopoietic stem cells. During terminal erythropoiesis, these cells incorporate large amounts of iron in order to generate hemoglobin and ultimately undergo enucleation to become mature red blood cells, ultimately delivering oxygen in the circulation. Thus, erythropoiesis is a finely tuned, multifaceted process requiring numerous properly timed physiological events to maintain efficient production of 2 million red blood cells per second in steady state. Iron is required for normal functioning in all human cells, the erythropoietic compartment consuming the majority in light of the high iron requirements for hemoglobin synthesis. Recent evidence regarding the crosstalk between erythropoiesis and iron metabolism sheds light on the regulation of iron availability by erythroblasts and the consequences of insufficient as well as excess iron on erythroid lineage proliferation and differentiation. In addition, significant progress has been made in our understanding of dysregulated iron metabolism in various congenital and acquired malignant and non-malignant diseases. Finally, we report several actual as well as theoretical opportunities for translating the recently acquired robust mechanistic understanding of iron metabolism regulation to improve management of patients with disordered erythropoiesis, such as anemia of chronic inflammation, β-thalassemia, polycythemia vera, and myelodysplastic syndromes.

## Introduction

Iron is an essential element for almost every organism on earth. Iron can donate and accept electrons from various substrates due to its unique oxidation-reduction properties, making it an important cofactor in mammalian cells. Despite its abundance in the Earth’s crust, high oxygen availability in the atmosphere leads to iron oxidation and the formation of poorly soluble ferric iron. As a consequence, iron-dependent organisms like mammals have evolved complex mechanisms to conserve and recycle iron, preventing its loss and enabling its enhanced absorption during a deficit or in times of increased demand. The physiological status of iron in biological systems can be delineated into three categories: iron coordinated by protein side chains, iron complexed within the porphyrin ring of heme, and iron within iron-sulfur clusters. Outside of these contexts, iron displays promiscuous reactivity that can damage cells and tissues poorly equipped for handling what is termed ‘labile iron.’ Extensive coordination is also required in multicellular organisms for movement of iron between organs, in and out of cells, and between compartments within cells to effectively avoid inadequate iron supply that may limit systemic functioning and excess iron that may be toxic to cells and tissues. Thus, dysregulated iron homeostasis can manifest as total body iron deficit (iron deficiency) or excess (iron overload), as well as iron maldistribution among tissues in which individual organs may be iron-deficient while other iron-overloaded. Such iron disorders may be caused by genetic lesions that directly impair iron regulation or conditions that impact iron regulation indirectly.

Although all cells in mammalian systems require small amounts of iron, systemic iron homeostasis is mainly regulated by the specific compartments involved in iron absorption, transport, storage and recycling, and high-level utilization. These include duodenal enterocytes; serum transferrin-bound iron; hepatocytes and macrophages in the liver and spleen; and erythroid precursors in the bone marrow, respectively. The largest of these compartments is the erythron, comprising the majority of total body iron in adult humans, mainly contained within hemoglobin inside erythroblasts and ultimately red blood cells (RBCs). Said another way, erythropoiesis, even at steady state, consumes most of the transferrin bound iron in circulation. Furthermore, stimulation of increased erythropoiesis (e.g. via exogenous erythropoietin [EPO], bleeding, phlebotomy, or hypoxia) requires enhanced iron availability to keep pace with acutely increased hemoglobin synthesis within erythroblasts in the bone marrow. A large body of observational work amassed in the last >50 y suggested the presence of a direct ‘erythroid regulator’ of iron metabolism to enable this crosstalk. The last few decades have provided a series of substantial advances, enabling significant progress in our understanding of iron metabolism regulation; normal, stress, and ineffective erythropoiesis; and the crosstalk between them. In addition, the development of novel tools, both experimental methods and models of disease, has furthered and provided additional opportunities to better understand these pathways, robustly confirming and extending previously held assumptions and conjectures. This article aims to bring together our collective understanding of what has been learned and developed to study iron metabolism and erythropoiesis in health and disease.

## Normal erythropoiesis

Erythropoiesis is a continuous process required for making new RBCs in order to replace the senescent RBCs lost at the end of their lifecycle. To determine how many RBCs are required, the bone marrow relies on the kidney in which interstitial fibroblasts in the renal medulla sense hypoxia, leading to the hypoxia-inducible factor 2 (HIF-2) mediated production and excretion of EPO. EPO binding to EPO receptor on erythroid precursors in the bone marrow in turn induces their survival, cell division, and differentiation to ultimately enucleate, producing reticulocytes that mature to RBCs in the circulation. Hemoglobin synthesis in the developing erythroblasts requires iron-containing heme.

The first wave of erythropoiesis starts as early as embryonic day 7.5 (E7.5) in mouse (Palis et al., 1999; Palis and Yoder, 2001; Baron et al., 2013) and 3 wk in human (Palis and Yoder, 2001; Dzierzak and Philipsen, 2013) in the yolk sac and produces large nucleated erythroid cells from hemangioblasts (Dzierzak and Philipsen, 2013; Palis, 2008; Baron et al., 2013). Due to its transient nature, it is termed ‘primitive’ erythropoiesis to distinguish it from ‘definitive’ erythropoiesis that gradually replaces primitive erythropoiesis in the fetal liver and bone marrow, persisting thereafter throughout life. Definitive erythropoiesis produces erythroid progenitor cells from hematopoietic stem cells (HSCs) that seed and differentiate within the fetal liver by ~E14.5 in the mouse (Palis et al., 1999; Palis and Yoder, 2001; Baron et al., 2013) and 7–8 wk in the human embryo (Palis and Yoder, 2001; Dzierzak and Philipsen, 2013). After birth, sites of erythropoiesis transition from liver to spleen and eventually bone marrow and enable robust RBC production needed to reach and sustain steady-state adult erythropoiesis levels by age 7 wk in the mouse (Chen et al., 2021).

**Figure 1.**
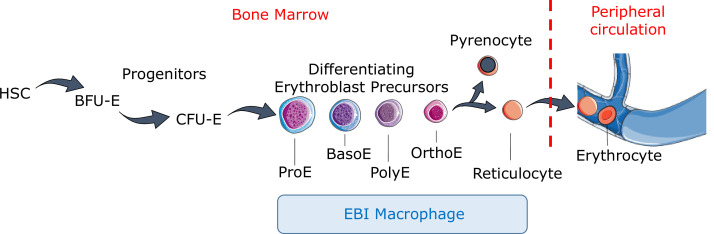
Definitive erythropoiesis. Definitive erythropoiesis in the adult organism is derived from hematopoietic stem cells (HSCs) with progressive movement of cells through three compartments: progenitors, erythroblast precursors, and erythrocytes. Erythroid progenitors (burst-forming unit-erythroid [BFU-E], colony-forming unit-erythroid [CFU-E]) are defined by their capacity to form colonies of maturing erythroid cells in vitro. Erythroid precursors are defined as pro-erythroblasts (ProE), basophilic erythroblasts (BasoE), polychromatophilic erythroblasts (PolyE), and orthochromatic erythroblasts (OrthoE) based on morphology and progressive change in the expression of surface markers. OrthoE enucleate to form a pyrenocyte, which contains the condensed nucleus, and a reticulocyte, which goes on to mature into an erythrocyte in the peripheral circulation. Erythroblast precursors undergo differentiation in contact with a macrophage within the erythroblastic island (EBI).

Definitive erythropoiesis is a multi-step, complex process that can be divided into three maturational stages, namely early-stage erythropoiesis, terminal erythroid differentiation, and reticulocyte maturation (Figure 1). Early-stage erythropoiesis consists of two erythroid progenitor stages, burst-forming unit-erythroid (BFU-E) and colony-forming unit-erythroid (CFU-E). BFU-E and CFU-E were initially defined by their ability to form distinct types of colonies of erythroid cells in semisolid media (Iscove and Sieber, 1975; Gregory and Eaves, 1977). With the development of flow cytometry technology in conjunction with the identification of surface markers, both murine and human BFU-E and CFU-E cells can now be identified and isolated by fluorescence-activated cell sorting (FACS) for subsequent cellular and molecular studies (Flygare et al., 2011; Li et al., 2014; Zhang et al., 2021). Furthermore, a recent study showed that human erythroid progenitor (EP) populations can be further subdivided into four subpopulations with EP1 representing predominantly BFU-E with EP2, EP3, and EP4 representing increasingly mature CFU-E populations with reduced proliferative responsiveness to stem cell factor (SCF), indicating heterogeneity of previously identified CFU-E population (Yan et al., 2021).

Terminal erythroid differentiation is a process by which proerythroblasts (Pro) differentiate sequentially to basophilic (Baso), polychromatic (Poly), and orthochromatic (Ortho) erythroblasts that expel their nuclei to become reticulocytes (Figure 1). During terminal erythroid differentiation, several notable changes occur, enabling one to selectively identify erythroblasts at distinct developmental stages based on morphological features. These changes include decreasing cell size, hemoglobin accumulation, chromatin condensation, and enucleation. Previously, Giemsa staining and morphological assessment by light microscopy were the only means for identifying at these terminally differentiating erythroblast stages. Currently, isolating and purifying both murine and human erythroblasts at distinct stages can be accomplished using surface markers and FACS analysis (Chen et al., 2009; Liu et al., 2013; Hu et al., 2013). These novel methods for isolating erythroid lineage cells at each stage have not only enabled the study of normal and disordered erythropoiesis in a stage-specific manner (Liu et al., 2013; Hu et al., 2013) but have also facilitated global molecular characterization of erythropoiesis. Various global omics analyses have been performed using purified erythroid cell populations (Li et al., 2014; An et al., 2014; Edwards et al., 2016; Schulz et al., 2019). Transcriptome analyses of human and murine erythroblasts reveal significant stage- and species-specific differences across stages of terminal erythroid differentiation (An et al., 2014). The epigenetic landscape of human erythropoiesis reveals that erythroid cells exhibit chromatin accessibility patterns distinct from other cell types. It also reveals stage-specific patterns of gene regulation (Schulz et al., 2019). Global omics analyses have also been performed on primitive erythroblasts during embryogenesis that revealed molecular similarities and differences between primitive and definitive erythropoiesis (Nemkov et al., 2022). The omic databases generated during the last decade not only contribute novel understanding of normal erythropoiesis regulation but also provide insight into disease pathophysiology and serve as rich resources for future studies.

Enucleation is a distinctive feature of mammalian erythropoiesis. Given the physiological significance of enucleation in generating highly deformable RBCs for effective oxygen delivery, understanding the mechanistic basis of enucleation has been an active area of investigation. Cytoskeleton proteins such as actin (Takano-Ohmuro et al., 1996; Ji et al., 2008; Watanabe et al., 2013; Ubukawa et al., 2020; Liu et al., 2021), tubulin (Chasis et al., 1989; Wang et al., 2012), myosin (Takano-Ohmuro et al., 1996; Ubukawa et al., 2012), tropomodulin (Sui et al., 2014), and chromatin condensation and lipid rafts (Ji et al., 2010; Konstantinidis et al., 2012; Malik et al., 2017; Zhao et al., 2019; Wang et al., 2021; Jeffery et al., 2021) are essential for normal enucleation. Although the function of actin and tubulin in enucleation suggests that erythroblast enucleation is a form of asymmetric cytokinesis, evidence documents that vesicle trafficking rather than cytokinesis is required for enucleation (Keerthivasan et al., 2010). This conclusion is supported by a recent finding that vesicle formation regulated by ERK/MAPK pathway mediates human erythroblast enucleation (An et al., 2021). Furthermore, although some evidence suggests that actin forms a contractile ring to help expel the nucleus (Ji et al., 2008), other data demonstrates that a dedicated cytoskeletal assembly in the cytoplasm, the ‘enucleosome,’ located contralateral to the site of enucleation, that is, at the rear of the nucleus, is most likely the driver of nuclear expulsion (Nowak et al., 2017). These findings indicate that despite relatively extensive studies on enucleation, the relevant mechanisms remain incompletely understood, somewhat controversial, and would benefit from further investigation.

Reticulocyte maturation is the final step of erythropoiesis. During this process, a series of major changes occur. These include membrane surface area loss via membrane vesiculation (Waugh et al., 1997), organelle (mitochondria and ribosome) clearance via autophagy (Gronowicz et al., 1984; Kundu et al., 2008; Zhang et al., 2009b), and membrane skeleton reorganization (Chasis et al., 1989; Liu et al., 2010; Liu et al., 2011). These changes together lead to fully functional mature RBCs with maximum hemoglobin carrying capacity and flexible yet stable membranes.

Erythropoiesis is tightly regulated by multiple soluble factors. To determine how many RBCs are required, the bone marrow relies on the kidney in which interstitial fibroblasts in the renal medulla sense hypoxia, leading to the HIF-2-mediated production and excretion of EPO. Very recent preliminary data using single-cell RNA and transposase-accessible chromatic (ATAC) sequencing to molecularly identify EPO-producing cells indicate that a distinct population of renal stromal cells, termed Norn cells, are the main source of EPO production in mice and humans (Kragesteen et al., 2023). EPO binding to EPO receptor (EPOR) on erythroid precursors in the bone marrow in turn induces their survival, cell division, and differentiation to ultimately produce RBCs. EPO and EPOR are indispensable for definitive erythropoiesis. Deletion of EPO or EPOR leads to embryonic lethality at approximately E13 due to severe anemia associated with defects in deﬁnitive erythropoiesis in mice (Wu et al., 1995). Other cytokines, growth factors, and hormones such as stem cell factor, interleukin-3, insulin-like growth factor 1, and glucocorticoids, although not essential for erythropoiesis, also promote proliferation of erythroid progenitors (Sonoda et al., 1988; Sonoda et al., 1994; Kolbus et al., 2003; Perry et al., 2007; Flygare et al., 2011). At the transcriptional level, red cell development is regulated by multiple transcription factors (Dzierzak and Philipsen, 2013; Andrieu-Soler and Soler, 2022), two of which, GATA1 and KLF1, are considered master regulators, indispensable for normal erythropoiesis (Pevny et al., 1995; Siatecka and Bieker, 2011).

Similar to EPO, iron is also essential for erythropoiesis. Hemoglobin synthesis in the developing erythroblasts requires iron-containing heme. While EPO allows survival of erythroid progenitors and early-stage erythroblasts by activating transcription of anti-apoptotic genes via the EPO/EPOR/JAK2/STAT5 signaling pathway (Witthuhn et al., 1993), iron modulates EPO responsiveness (Khalil et al., 2018) and is also essential for differentiation of early- to late-stage erythroblasts, during which time iron incorporation into the protoporphyrin ring is required as the last step in heme synthesis. EPO can thus be regarded as a ‘driver’ of erythropoiesis, while iron acts as a ‘modulator’ and also serves as the fuel for the production of RBCs. In addition, recent data demonstrates that iron may be involved in the modulation of EPO responsiveness via the regulatory function of monoferric transferrin.

## Physiological regulation and dysregulation of iron metabolism

### Systemic regulation of iron metabolism

**Figure 2.**
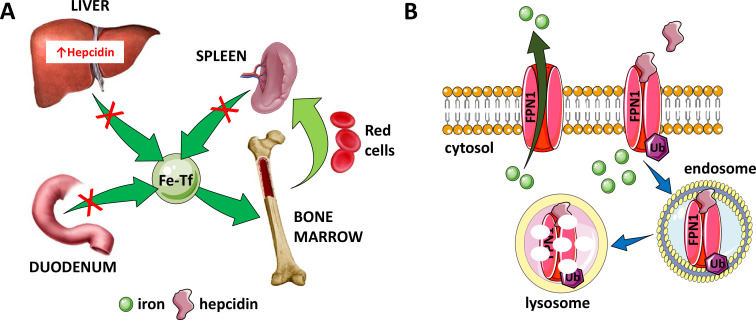
Hepcidin is central to the regulation of iron metabolism. (**A**) Systemically, hepcidin is a negative regulator of iron flows such that increased hepcidin synthesis (which mainly occurs in the liver) leads to hypoferremia by decreasing iron absorption in the duodenum, iron recycling from splenic macrophages, and iron release from hepatocyte stores. (**B**) The mechanism of action of hepcidin involves binding to and occluding ferroportin, induction of ferroportin ubiquitination, followed by endocytosis and lysosomal degradation of the ferroportin:hepcidin complex. Fe-Tf, transferrin-bound iron; FPN1, ferroportin1; Ub, ubiquitination.

**Figure 3.**
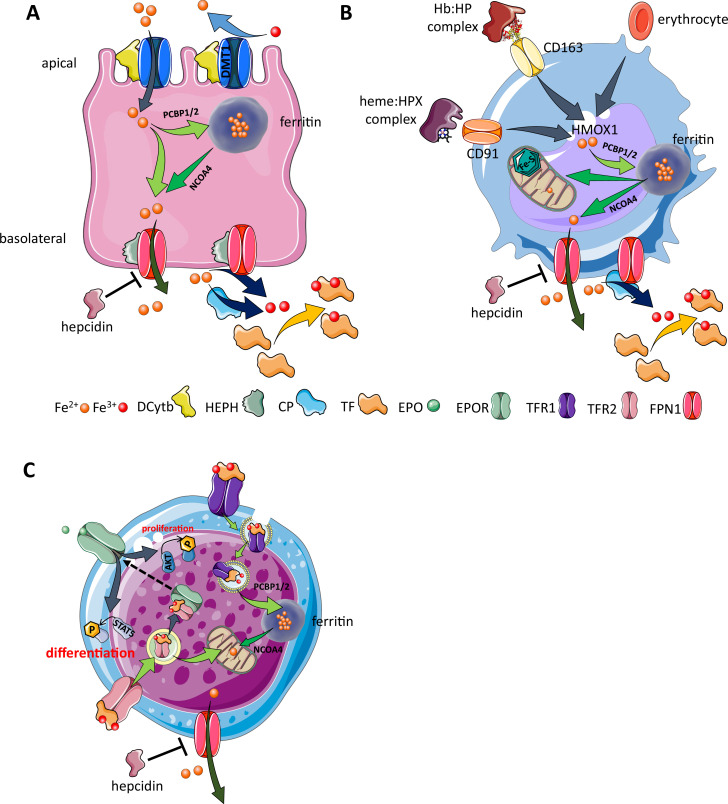
Cellular iron metabolism. Intracellular iron homeostasis is balanced by coordinated iron uptake, utilization, storage, and export. The three main cells of interest include duodenal enterocytes (involved in systemic iron absorption), reticuloendothelial macrophages (involved in systemic iron recycling), and erythroblasts (main location of systemic iron utilization for hemoglobin synthesis during erythropoiesis). (**A**) *Duodenal enterocyte*: absorbed inorganic ferric iron (Fe^3+^) must be first converted to ferrous iron (Fe^2+^) via ferrireductase Dcytb and subsequently taken up by iron importer DMT1. Once inside the cell, iron is shuttled to ferritin via iron chaperones PCBP1/2 and stored there or shuttled out of ferritin by NCOA4 for export via FPN1. During iron export, Fe^2+^ must be oxidized to Fe^3+^ by HEPH or CP and loaded onto TF for transport in the circulation. Hepcidin prevents iron export at the basolateral cell membrane and results in ferritin iron accumulation within the enterocyte. (**B**) *Macrophage*: splenic and liver macrophages are specifically equipped with mechanisms to enable direct erythrophagocytosis, uptake of Hb:HP complexes via CD163, and heme:HPX complexes via CD91. The heme extracted from these pathways is processed by HMOX1 to liberate iron that is then either incorporated into ferritin or exported from the cell via FPN1 and loaded onto TF for delivery to iron-requiring cells. (**C**) *Erythroblast*: iron-loaded TF binds to TFR1 on the surface of cells with erythroblasts expressing the highest concentration of TFR1 relative to other cells in light of their high iron requirements. These complexes localize to clathrin-coated pits that invaginate to form specialized endosomes where proton pumps decrease the pH and transported Fe^3+^ is reduced by STEAP3 for export from the endosome via DMT1. Erythroblasts shuttle much of their iron to the mitochondria by an incompletely understood mechanism where it is incorporated into protoporphyrin. FPN1 is also expressed on erythroblasts but purpose of iron export in erythroblasts is incompletely understood. Finally, iron loaded TF also binds TFR2, which is thought to function as an iron sensor to coordinate iron supply with erythropoietic output by modulating EPOR localization and consequently EPO responsiveness; a detailed mechanistic understanding of TFR2’s role in erythropoiesis (DMT1, divalent metal transporter 1; Dcytb, duodenal cytochrome B reductase; FPN, ferroportin 1; HEPH, hephaestin; CP, ceruloplasmin; TF, transferrin; Fe^3+^, ferric iron; Fe^2+^, ferrous iron; Hb, hemoglobin; HP, haptoglobin; HPX, hemopexin; CD91 and 169, cluster of differentiation 91 and 163; HMOX1, heme oxygenase 1; TFR1 and 2, transferrin receptor 1 and 2; EPO, erythropoietin; EPOR, EPO receptor; PCBP1, poly(rC)-binding protein 1; NCOA4, nuclear receptor coactivator 4; pSTAT5, phosphorylated signal transducer and activator of transcription 5; pAKT, phosphorylated protein kinase B).

Because of its low bioavailability, complex living organisms have developed sophisticated mechanisms to obtain, distribute, and sequester iron that have also enabled competition for iron with pathogens and prevention of iron excess. In a seminal discovery more than two decades ago, the peptide hormone hepcidin, secreted primarily by hepatocytes, has been shown to be the principal regulator of iron homeostasis (Krause et al., 2000; Park et al., 2001; Ganz, 2005), modulating dietary iron absorption, iron recycling by macrophages, and the release of iron from hepatic stores (Figure 2A). Hepcidin is a negative regulator of iron flows with high hepcidin concentration typically resulting in the blockade of iron absorption and sequestration of cellular iron. Hepcidin downregulates iron release into plasma by binding to and functionally downregulating ferroportin 1, the sole exporter of intracellular iron (Nemeth et al., 2004; Donovan et al., 2005; Figure 2B). Ferroportin is evolutionarily conserved and is found in microbes, invertebrates, plants, and animals (Taniguchi et al., 2015). In humans, ferroportin is found in duodenal enterocytes, macrophages, and hepatocytes, all cells involved in iron transport (Figure 3). In addition, erythroid progenitors and precursors in the bone marrow as well as circulating RBCs also express ferroportin (Zhang et al., 2018; Figure 3C), an interesting finding that remains incompletely understood.

Hepcidin:ferroportin binding leads to both occlusion of the ferroportin channel (Aschemeyer et al., 2018) and induction of a conformational change, leading to ferroportin ubiquitination, endocytosis of the complex (Qiao et al., 2012), and its ultimate lysosomal degradation (Nemeth et al., 2004; Figure 2B). More recent work provides further structural and functional detail of the hepcidin:ferroportin interaction using cryogenic electron microscopy, identifying the 80-fold enhanced hepcidin binding to iron-loaded ferroportin and elucidating targeted ferroportin degradation in the presence of iron (Billesbølle et al., 2020). From a systemic perspective, this block in cellular iron efflux leads to circulating hypoferremia as a consequence of continued iron uptake from the circulation, leading to iron consumption if it is not replaced by iron efflux from enterocytes, macrophages, and hepatocytes. Thus, maintaining a stable supply of iron in the circulation is dependent on hepcidin-mediated post-translational regulation of ferroportin. In addition, ferroportin expression in erythrophagocytosing macrophages is also transcriptionally regulated by heme (Marro et al., 2010) and under translational regulation (i.e. iron response elements on messenger RNA bound to iron response proteins) by mechanisms independent of hepcidin regulation (Zhang et al., 2009a). Finally, recent work also provides evidence that transferrin also interacts with ferroportin, leading to ferroportin internalization and degradation by a well-established pathway, and that only extra-physiological levels of hepcidin interfere with the transferrin:ferroportin interaction (Baringer et al., 2023). The full physiological significance of these finding remains to be determined.

Ferroportin expression on the basolateral side of duodenal enterocytes, on splenic and liver macrophages, and on hepatocytes enables hepcidin regulation of iron absorption, recycling, and storage, respectively (Figure 3A and B). Because hepcidin is a negative regulator of iron metabolism, decreased hepcidin concentration results in increased iron absorption and increased release of iron from intracellular compartments in hepatocytes and macrophages, enabling recovery from iron deficiency. To elucidate, low hepcidin levels result in greater ferroportin activity on duodenal enterocytes, leading to the depletion of enterocyte iron levels with consequently decreased activity of oxygen- and iron-dependent prolyl hydroxylases that target hypoxia-inducible factors (HIFs) for degradation in proteasomes, stabilizing HIF. HIF2α is an important local regulator of transcription of the apical iron importer divalent metal transporter 1 (DMT1), the iron reductase duodenal cytochrome *b* (DCYTB), and the basolateral exporter ferroportin (Schwartz et al., 2019). Taken together, HIF2α stabilization coordinates apical import of dietary iron with the hepcidin-controlled activity of ferroportin to enhance absorption of intestinal iron in iron-deficient conditions.

In some pathological conditions, insufficiently increased hepcidin results in excessive iron released into the circulation, overwhelming transferrin’s iron binding capacity, resulting in the generation of non-transferrin bound iron (NTBI) (Esposito et al., 2003). NTBI, in particular its redox-active form, labile plasma iron (LPI), is thought to be the pathogenetic driver of clinically significant iron overload in diseases of primary and secondary hemochromatosis (Cabantchik et al., 2005). NTBI/LPI is unavailable for erythropoiesis, is taken up by non-hematopoietic cells in a dysregulated manner, causes parenchymal iron deposition (Jenkitkasemwong et al., 2015), and can result in free radical damage to cells and organs, leading to the morbidity and mortality of iron overload. More detailed pathophysiology of iron overload is beyond the scope of the current review; an excellent review of hepcidin in disorders of iron regulation was recently published (Nemeth and Ganz, 2023).

### Cellular regulation of iron metabolism

As mentioned, iron is required for homeostatic function in all cells, essential for the production of heme and iron-sulfur clusters, themselves components of proteins/enzymes involved in respiration, nucleic acid replication and repair, metabolic reactions, and host defense. Specifically, iron is necessary for enzymatic reactions in the electron transport chain and the tricarboxylic acid cycle, and iron participates in reactions catalyzed by microsomal cytochromes involved in the detoxification of drugs and other foreign substances. Despite broad functioning in physiological processes, the majority of iron functions as an oxygen carrier in the heme groups of hemoglobin and myoglobin molecules. Because iron can be highly toxic to cells, cellular iron trafficking requires deliberate coordination to enable its safe utilization. Here, we focus on specific cell types that are central to systemic iron metabolism.

1. *Duodenal enterocytes*: The primary site of dietary iron absorption involves enterocytes within duodenal villi. These polarized cells are in contact with the gut lumen and dietary contents on their apical side and with blood in circulation on their basolateral side (Figure 3A). Non-heme iron is imported from the lumen by the apical enterocyte DMT1 (Gunshin et al., 1997; Fleming et al., 1997), a metal transporter that takes up iron after iron reduction from ferric (Fe^3+^) to ferrous (Fe^2+^) state (McKie et al., 2001). Iron that is not used for enterocyte function is either stored within ferritin or exported by ferroportin on the basolateral surface to be loaded onto transferrin in circulation. As transferrin-bound iron is obligate Fe^3+^, intracellular Fe^2+^ must first be oxidized at the basolateral surface to enable its export (Vulpe et al., 1999). Finally, iron that is not exported at the enterocyte’s basolateral surface into the circulation is lost during mucosal shedding. The concentration of hepcidin in circulation regulates iron absorption at the basolateral surface of the enterocyte such that low hepcidin levels correlate with increased iron absorption to enable recovery from iron deficiency while high hepcidin levels prevent additional iron absorption (Figure 3A).
2. *Reticuloendothelial macrophages*: At the end of their 120-day life cycle, RBC recycling by macrophages in the spleen and liver supports the recovery of iron for further systemic use (Figure 3B). In addition, RBC hemolysis leads to the release of hemoglobin into the circulation, where it is bound by haptoglobin, and circulating heme:hemopexin complexes, taken up by macrophages via CD163 and CD91, respectively. Macrophages are equipped with mechanisms to recover iron from heme from both intact and hemolyzed RBCs via heme oxygenase 1 (HMOX1) (Figure 3B). As in duodenal enterocytes, depending on systemic requirements, a fraction of the recovered iron is stored in macrophages, bound for intracellular storage in cytosolic ferritin with the rest exported via ferroportin back into the circulation to bind transferrin when needed to maintain equilibrium of iron flux or when erythropoiesis is increased (Figure 3B).
3. *Erythroblasts*: The vast majority of transferrin-bound iron in the circulation is targeted to developing erythroid progenitors in the bone marrow to eventually be incorporated into heme (Finch et al., 1970). Iron uptake for erythropoiesis is the result of transferrin:transferrin receptor 1 (TFR1) binding (Figure 3C). Transferrin protein, with two iron molecules (also known as holo-transferrin or diferric transferrin), binds with high affinity to TFR1 at physiological pH 7.4; apo-transferrin (lacking iron) does not. Furthermore, monoferric transferrins, which bind TFR1 with affinity intermediate between holo- and apo-transferrin, are the most abundant transferrin moieties in the circulation (Luck and Mason, 2012; Parrow et al., 2019; Klausner et al., 1983a; Iacopetta et al., 1983; Klausner et al., 1983b; Dautry-Varsat et al., 1983; van Renswoude et al., 1982). A second transferrin receptor, TFR2, has been shown to mediate signaling events unrelated to meeting cellular iron needs, but a detailed understanding of its role in the crosstalk between erythropoiesis and iron metabolism awaits additional hypothesis-driven evaluation.
4. *Hepatocytes*: Hepatocytes account for 60–80% of liver cell mass, are metabolically active cells with numerous mitochondria, are responsible for carbohydrate metabolism, and contribute to a wide range of regulatory proteins, vitamins, and hormones required for local and systemic function. As such, hepatocytes are the main source of hepcidin synthesis and secretion. As a consequence, the central function of these cells for the purposes of this article is the mechanistic regulation of hepcidin.

## Physiological regulation of hepcidin expression

**Figure 4.**
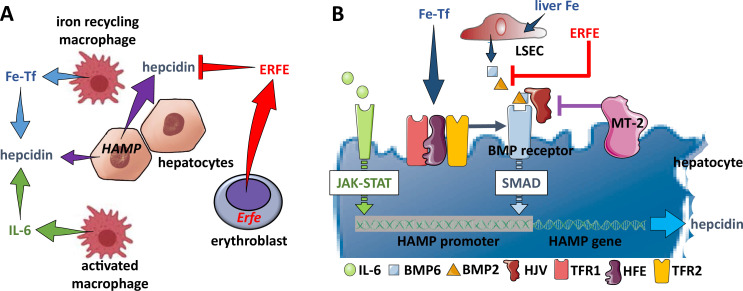
Hepcidin regulation. (**A**) Systemically, hepcidin is stimulated by transferrin-bound iron in the circulation and liver iron stores as well as by systemic inflammation and suppressed by enhanced erythroid activity. (**B**) Regulation of hepcidin expression in the hepatocyte involves JAK-STAT signaling as a consequence of IL-6 receptor stimulation and SMAD signaling as a consequence of a BMP receptor complex stimulation. IL-6 and BMP2/6 binding their receptors, respectively, leads to stimulation of hepcidin expression. Stimulation of erythropoiesis leads to the expression and secretion of erythroferrone that sequesters BMP2/6 to suppress SMAD signaling, decreasing hepcidin. Additional coregulation via matriptase-2 and hemojuvelin as well as systemic iron sensing by TFR1, HFE, and TFR2 enhance BMP receptor stimulation and increase hepcidin expression. Fe-Tf, transferrin-bound iron; IL-6, interleukin 6; HAMP, gene name for hepcidin; ERFE, erythroferrone; LSEC, liver sinusoidal endothelial cell; TFR1/2, transferrin receptor 1 and 2; HFE, homeostatic iron regulator; HJV, hemojuvelin; MT-2, matriptase 2; JAK-STAT, janus kinase and signal transducer and activator of transcription; SMAD, small mothers against decapentaplegic.

Hepcidin expression is predominantly regulated by iron, inflammation, and erythropoiesis (Figure 4A). First, *HAMP* transcription in hepatocytes is upregulated by iron loading and suppressed by iron deficiency as well as expanded or ineffective erythropoiesis. The regulation of hepcidin by iron is incompletely understood. Murine models suggest hepatocytes sense local and systemic iron status by binding bone morphogenetic proteins (BMPs), primarily BMP6, BMP2, and/or their heterodimers via BMP receptors, and transferrin-bound iron via TFR1 and TFR2, leading to signaling to induce hepcidin expression (Camaschella et al., 2020; Figure 4B). Second, hepatocyte hepcidin expression is mediated indirectly by iron in response to iron-induced BMP production by liver sinusoidal endothelial cells (LSECs) (Enns et al., 2013). Recent evidence demonstrates that BMP6 expression in primary LSEC ex vivo is induced in response to iron only when co-cultured with primary hepatocytes or supernatants from primary hepatocyte cultures (Colucci et al., 2022). Additional recent effort provides evidence for a minor functional role of LSEC TFR1-mediated iron uptake and BMP6 induction in iron limited conditions and TFR1-independent iron-mediated regulation of LSEC BMP6 expression in iron-rich conditions (Fisher et al., 2022). Although how BMP expression is induced in LSEC is not fully understood, the BMP pathway is critical for hepcidin the regulation by iron (Truksa et al., 2006; Babitt et al., 2007). Specifically, BMP6 and BMP2 binding hepatocellular BMP receptor triggers phosphorylation and signaling via SMAD1/5/8 that, coupled with SMAD4, translocate to the nucleus to induce *HAMP* expression (Figure 4B). Third, several hepatocellular surface molecules modulate *HAMP* activation in response to iron status, enabling hepatocytes to directly sense iron via expression of TFR1, TFR2, and HFE. To briefly delineate, HFE association with TFR1 under low iron conditions is displaced when TFR1 binds monoferric or diferric transferrin (Feder et al., 1998; Bennett et al., 2000; Giannetti and Björkman, 2004; Lebrón et al., 1998). Although a mechanistic understanding of how TFR2 contributes to hepcidin regulation remains unclear, we surmise that as serum iron concentration increases, increased transferrin:TFR2 binding induces TFR2 membrane stabilization (Johnson and Enns, 2004; Robb and Wessling-Resnick, 2004) and possibly HFE binding to TFR2. This HFE:TFR2 complex interacts with hemojuvelin (HJV), the iron-specific BMP co-receptor, and potentiates the BMP signaling pathway to *HAMP* expression (Figure 4B). Furthermore, the specific mechanism by which HJV regulates hepcidin is incompletely understood. For example, recent evidence demonstrates that HJV interaction with neogenin, a ubiquitously expressed transmembrane protein, is required for hepcidin regulation (Enns et al., 2021). Finally, the pathway is negatively regulated by the transmembrane serine protease matriptase 2 (i.e. TMPRSS6), which by binding HJV, BMP receptor, and/or HFE decreases signaling to *HAMP* expression (Enns et al., 2020). Thus, both TFR2 and HFE:TFR1 complex function as the main iron sensors (Schmidt et al., 2008; Robb and Wessling-Resnick, 2004) and communicate systemic iron status to modify hepatocyte hepcidin production and secretion with multiple co-factors modulating this signal.

Interestingly, hepatocyte TFR1 also influences systemic iron homeostasis by interacting with the hemochromatosis protein HFE to regulate hepcidin production (Fillebeen et al., 2019; Xiao et al., 2023). Although loss of hepatic *Tfrc* is not associated with grossly altered iron metabolism, hepatocyte-selective *Tfrc* knockout mice show predisposition to anemia making their unchanged hepcidin levels inappropriately high relative to serum and liver iron concentrations and ERFE levels. In addition, ablation of hepatocyte *Tfrc* does not modify the iron phenotype in *Hfe* knockout mice. Lack of *Tfrc* also ameliorates hepcidin deficiency and liver iron loading.

## Crosstalk between erythropoiesis and iron metabolism

Hemoglobin synthesis in erythroblasts requires large amount of iron, providing a strong rationale for erythropoiesis-mediated regulation of iron availability. For instance, stimulated erythropoiesis (e.g. in response to bleeding, repeated or large volume phlebotomy, hypoxia, or administration of exogenous EPO) leads to increased iron absorption, and the last few decades have provided a more robust mechanistic understanding of how iron availability is regulated by erythropoiesis. For the purposes of this article, we will discuss several aspects of this crosstalk, including how iron is taken up and chaperoned in erythroblasts, how erythropoiesis modulates iron metabolism directly and indirectly, and how iron metabolism itself impacts erythropoiesis.

### Iron uptake and trafficking in erythroblasts

Hemoglobin, both in circulation and within the bone marrow, contains more than two-thirds of the body’s iron, and the majority of circulating iron is destined for uptake by erythroblasts (Finch et al., 1970). Iron uptake for erythropoiesis occurs via transferrin binding to TFR1 (Figure 3C). Transferrin bound to TFR1 is internalized as a complex by receptor-mediated endocytosis (Klausner et al., 1983a; Iacopetta et al., 1983), which is coordinated with endosomal acidification, resulting in the release of iron from transferrin (Klausner et al., 1983b; Dautry-Varsat et al., 1983; van Renswoude et al., 1982). Several hypotheses have been tested to ascertain how iron is transported within cells, the most compelling of which involves the cytosolic chaperone Poly(rC)-binding protein 1 (PCBP1). PCBP1 delivers iron to ferritin (Leidgens et al., 2013; Ryu et al., 2017; Figure 3C). Evidence from *Pcbp1* knockout mice, with microcytosis and anemia, demonstrate that iron delivery to ferritin is required for normal erythropoiesis (Ryu et al., 2017). In addition, PCBP2 is also required for ferritin complex formation (Leidgens et al., 2013). Furthermore, an autophagic process to extract iron from the ferritin core is mediated by nuclear receptor coactivator 4 (NCOA4), a selective cargo receptor for autophagic ferritin turn-over, critical for regulation of intracellular iron availability (Mancias et al., 2014; Dowdle et al., 2014; Figure 3C). In iron-replete states, PCBP1 and PCBP2 expression is enhanced while NCOA4 is targeted to the proteasome for degradation (Mancias et al., 2015). This process, termed ferritinophagy, is believed to provide iron to the mitochondria, the main organelle involved in heme and hemoglobin synthesis during erythropoiesis. Alternatively, or in concert, the ‘kiss-and-run’ model may support the of transfer iron without chaperones when transferrin iron containing endosomes and mitochondria come into contact with one another (Hamdi et al., 2016). Finally, transferrin-bound iron internalized by TFR2 may undergo trafficking to lysosomes and subsequent transfer to mitochondria via Mucolipin 1 and Mitofusin 2 (Khalil et al., 2017; Figure 3C). Taken together, despite important recently uncovered mechanistic findings, the nuances of how iron trafficking in erythroblasts is dysregulated and contributes to disordered erythropoiesis are incompletely understood.

### Erythropoiesis-mediated regulation of iron metabolism

During the last century, investigators have proposed that an erythroid regulator strongly influences iron homeostasis. The discovery of hepcidin as an iron-regulatory hormone heralded a new era in exploring the mechanistic foundation of an erythroid regulator of iron homeostasis. For instance, stimulation of erythropoiesis—by bleeding, anemia, hypoxia, or injection of exogenous EPO—strongly suppresses hepcidin production in mice and humans, and iron absorption increases, often dramatically, during such stress erythropoiesis to accommodate increased iron demand. Initial exploration of EPO itself as a hepcidin suppressor revealed a lack of direct effect in in vitro studies in isolated liver cells (Gammella et al., 2015), implicating an intermediary EPO-responsive suppressor of hepcidin.

To explore the mechanism(s) underlying erythropoiesis-mediated regulation of hepcidin required separating how EPO, hypoxia, anemia, reticulocytosis, and erythropoiesis itself are individually involved. Prior experiments demonstrate that phlebotomy, EPO administration, and hemolysis all resulted in decreased hepcidin expression (Nicolas et al., 2002a; Nicolas et al., 2002b; Vokurka et al., 2006). Additional studies revealed that bone marrow ablation prevents hepcidin suppression in response to phlebotomy, EPO administration, and hemolysis (Vokurka et al., 2006; Pak et al., 2006), strongly supporting the hypothesis that erythroid regulation of hepcidin is a consequence of expanded, stress, or ineffective erythropoiesis. Consistently, iron-loading anemias exhibit complicated crosstalk between erythropoiesis and iron metabolism that remain incompletely understood. Such diseases of concurrent iron overload and expanded or ineffective erythropoiesis (e.g. β-thalassemia, some cases of myelodysplastic syndromes [MDS], and dyserythropoietic anemias) exhibit lower-than-expected hepcidin expression, insufficiently elevated relative to increased iron stores. In fact, insufficiently elevated hepcidin expression results in iron overload in these diseases, providing further support to the hypothesis that an ‘erythroid factor’ regulates iron metabolism (Ginzburg et al., 2009; Gardenghi et al., 2007).

Such an erythroid factor secreted by erythroid precursors, functioning as a hormone to distally suppress hepcidin expression in the liver, was predicted several decades prior to its recent discovery. Although multiple factors correlate with pathologically expanded or ineffective erythropoiesis, they do not support physiological regulation of iron by erythropoiesis. For example, although circulating growth differentiation factor 15 (GDF15) increases in patients with some congenital and acquired anemias and inversely correlates with hepcidin (Tanno et al., 2007), levels of GDF15 and hepcidin correlate poorly in phlebotomized mice (Casanovas et al., 2013) and in MDS patients (Santini et al., 2011), suggesting that mechanisms of hepcidin suppression by erythropoiesis may be disease specific. Furthermore, hypoxia has been shown to decrease hepcidin expression by a novel regulatory pathway exerted via platelet-derived growth factor BB (PDGF-BB), leading to increased availability of circulating iron that can be used for erythropoiesis (Sonnweber et al., 2014). To interrogate whether PDGF-BB is directly regulated by erythropoiesis, mice were treated with EPO demonstrating no significant impact on serum PDGF-BB concentration. Additional evaluation of this mechanism of regulation is needed to understand its full impact and contribution to physiological and/or pathophysiological hepcidin regulation.

Finally and importantly, the discovery of erythroferrone (ERFE) provided a mechanism for the physiological regulation of hepcidin in the absence of chronic disease (Kautz et al., 2014). *ERFE* is expressed in bone marrow erythroblasts (Figure 4A). As *Erfe*^-/-^ mice exhibit only mild anemia during the postnatal period (Kautz et al., 2014), ERFE expression increases post-phlebotomy and in response to exogenous EPO, supporting a hypothesis that its main function is to facilitate iron mobilization during recovery from transient anemia. Consistently, hepcidin suppression is dampened in *Erfe*^+/-^ and abrogated in *Erfe*^-/-^ mice (Kautz et al., 2014) after phlebotomy. An evaluation of the mechanism of ERFE’s regulation of hepcidin demonstrates that ERFE sequesters BMP2 and BMP6, resulting in decreased BMP:BMPR binding, decreased BMP:SMAD signaling, and decreased hepcidin expression (Arezes et al., 2018; Wang et al., 2020; Figure 4A and B), increasing iron absorption and release from intracellular iron stores to meet the iron requirements of temporarily expanded erythropoiesis during recovery from transient anemia. However, additional regulators may also exist in light of some persistent hepcidin suppression in phlebotomized ERFE knockout mice and ongoing iron accumulation in β-thalassemic ERFE knockout mice (Kautz et al., 2014; Kautz et al., 2015).

### Iron-mediated regulation of erythropoiesis

Anemia as a result of systemic iron deficiency is the most common cause of anemia worldwide. There is great consensus that iron deficiency inhibits the production of heme and hemoglobin but is erroneously synonymous with the resultant anemia. However, decreased heme and hemoglobin production in iron-deficient conditions contributes to decreased mean corpuscular volume (MCV) and hemoglobin (MCH). Conversely, disease states of excess iron are often associated with higher MCV and MCH as a functional utilization of iron within a non-toxic compartment (McLaren et al., 2007). Anemia, on the other hand, occurs when iron availability decreases below a threshold, impeding the maturation of erythroblasts and thus decreasing production of RBCs. Recent data provides mechanistic evidence of what is termed the ‘iron restriction response,’ demonstrating regulation of erythroid precursor differentiation during iron deficiency (Bullock et al., 2010; Khalil et al., 2018). The proposed mechanisms involve mitochondrial aconitase enzymes, TFR2, and scribble-mediated EPO receptor regulation, as well as effects on the erythroblast cell cycle (Talbot et al., 2011; Khalil et al., 2018) that converge on the decreased EPO-responsiveness of erythroblasts.

Specifically, recent studies reveal a novel iron-sensing function of TFR2 in erythropoiesis, via its interaction with EPOR (Forejtnikovà et al., 2010; Nai et al., 2015; Lee et al., 2012; Rishi et al., 2016; Fouquet et al., 2021; Figure 3C). However, the effect of TFR2 on EPO sensitivity remains incompletely understood. While studies in cell culture systems suggest that TFR2 increases EPO sensitivity by enhancing cell surface EPOR and downstream signaling (Forejtnikovà et al., 2010; Fouquet et al., 2021), mice with TFR2 knockout in the bone marrow demonstrate an increase, rather than the predicted decrease, in EPO sensitivity—but only during iron deficiency (Rishi et al., 2016). Likewise, iron-deficient mouse chimeras with *Tfr2*-deficient hematopoietic cells demonstrate increased EPO sensitivity, including erythrocytosis and activation of the JAK2-STAT5 and AKT pathways (Nai et al., 2015). Mechanisms by which erythroid TFR2 may regulate EPO sensitivity have not been completely delineated. Recent evidence identified a role for TFR2 in modulating surface EPOR delivery in response to iron availability. Specifically, erythroid iron restriction accelerates TFR2 trafficking to the lysosome and enhances catabolism of TFR2-Scribble complexes. The resultant deficiency of Scribble leads to diminished surface delivery of EPOR vesicles and diminished EPO responsiveness (Khalil et al., 2018). These findings suggest that manipulating TFR2 catabolism could provide a therapeutic approach to erythropoietic disorders with aberrant EPO responsiveness. We anticipate that the effect of TFR2 on EPO sensitivity depends upon the relative transferrin forms available for binding in the circulation and therefore reflect systemic iron status on erythropoiesis (Parrow et al., 2019).

Recent studies have also implicated loss of ferritin-induced stabilization of the microtubule cytoskeleton as a contributor to the erythroid iron restriction response, possibly explaining the misshapen RBCs, poikilocytes, characteristic of iron deficiency anemia (Goldfarb et al., 2021). Finally, evidence points to the importance of transferrin not only in iron delivery for hemoglobin synthesis but in regulation of erythroblast differentiation. As noted above, transferrin can be found in the circulation as holo-transferrin or diferric transferrin, monoferric transferrin, or apo-transferrin. Monoferric transferrins, either monoferric N (monoN) or monoferric C (monoC) lobe transferrin, are the most abundant transferrin moieties in the circulation (Luck and Mason, 2012; Parrow et al., 2019; Klausner et al., 1983a; Klausner et al., 1983b; Iacopetta et al., 1983; Dautry-Varsat et al., 1983; van Renswoude et al., 1982). Interestingly, relative distribution of the monoferric transferrin forms varies with iron status, such that the ratio of monoN to monoC transferrin decreases as serum iron falls (Pagani et al., 2019; Porter, 2020; Welch, 1992). To investigate the potential effects of transferrin lobe-specific iron occupancy, mice in which iron-binding was blocked from binding to either the N or the C lobe of transferrin (Gomme et al., 2005) were generated. These mice exhibit important differences from each other in both iron metabolism and erythropoiesis. Specifically, monoC-blocked mice predominantly have circulating monoN transferrin and demonstrate enhanced EPO sensitivity and hepcidin responsiveness to iron compared with monoN-blocked mice in which monoC transferrin is predominantly found in the circulation (Parrow et al., 2019). Conversely, primary disease states of excess iron are often associated with expanded RBC size and higher cellular hemoglobin concentrations as a functional utilization of iron within a non-toxic compartment (McLaren et al., 2007). Taken together, how iron regulates erythropoiesis remains substantially but incompletely understood.

## Mechanisms underlying anemia related to iron metabolism

Although there is a broad range of anemia-causing mechanisms, we will focus in this review on causes related to iron metabolism. In addition to iron deficiency anemia, these foremost include anemia of chronic inflammation (ACI). In addition, iron refractory iron deficiency anemia (IRIDA) is an interesting albeit rare form of IDA discussed here. In a subsequent section below, we will also discuss iron metabolism in the context of ineffective erythropoiesis in iron loading anemias, namely β-thalassemias and MDS.

### Anemia of chronic inflammation

**Figure 5.**
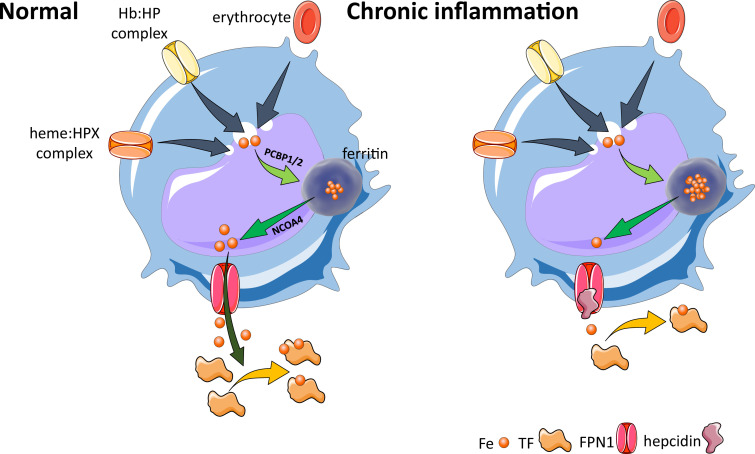
Effects of inflammation on iron recycling. Under normal conditions, iron recycling from multiple sources within macrophages leads to export of iron via ferroportin back into the circulation where it is loaded onto transferrin and delivered to cells with iron requirements (e.g. for hemoglobin synthesis in erythroblasts during erythropoiesis in the bone marrow). Increased hepcidin in states associated with chronic inflammation lead to binding to and occlusion of the ferroportin channel, preventing iron egress from cells involved in iron recycling (e.g. splenic red pulp macrophages), leading to the accumulation of iron within cellular ferritin core, and causing decreased iron-bound transferrin (low transferrin saturation). This decreased availability of iron for erythropoiesis results in anemia of chronic inflammation. Fe, iron; FPN, ferroportin 1; TF, transferrin; Hb, hemoglobin; HP, haptoglobin; HPX, hemopexin; PCBP1, poly(rC)-binding protein 1; NCOA4, nuclear receptor coactivator 4.

ACI is also termed anemia of chronic disease; while iron deficiency results in sometimes severe (i.e. hemoglobin 5–7 g/dL) microcytic, hypochromic anemia, ACI is typically a milder normocytic normochromic hypoproliferative anemia (i.e. hemoglobin 8–10 g/dL) and is considered the second most frequent anemia in the world, after IDA (Weiss and Goodnough, 2005). Both present with decreased circulating serum iron concentration and transferrin saturation, but while IDA is characterized by anemia with depleted iron stores (i.e. serum ferritin below the lower limit of normal), iron stores are ample in ACI. In the setting of inflammation, differentiating ACI from iron deficiency anemia may be challenging, and iron deficiency anemia may co-exist with ACI (Bressman et al., 2021). ACI is also the most common anemia in hospitalized patients, found in conditions associated with an activated immune response, including chronic infections, autoimmune and inflammatory illnesses and malignancy. The underlying cause of anemia in these diseases is multifactorial, resulting from the effects of inflammatory cytokines, particularly interleukin-1 (IL-1), IL-6, IL-10, tumor necrosis factor-α (TNF-α), interferon-γ (IFN-ɣ), IFN-α, and IFN-β, all or some of which are increased in most inflammatory processes (Raj, 2009). Multiple lines of evidence suggest that elevated inflammatory cytokines lead to increased iron sequestration and resultant decreases in iron availability for erythropoiesis and hemoglobin synthesis, directly and indirectly inhibiting erythroid progenitor differentiation, and resulting in a decreased EPO-responsiveness to anemia (Figure 5). The mechanism resulting in anemia in ACI is similar to that of IDA when iron stores are depleted (due to poor iron absorption alone or insufficiently enhanced in the setting of bleeding) as both conditions lead to decreased iron availability for erythropoiesis. The decreased iron availability may also act in a synergistic manner with the inflammatory cytokines in ACI, potentiating their capacity for direct suppression of erythroblasts (Richardson et al., 2013).

Previously considered a diagnosis of exclusion, with treatment of ACI mainly focused on the underlying disease, the identification of the peptide hormone hepcidin and its major role in the pathophysiology of ACI have enabled both an enhanced mechanistic understanding and development of novel therapeutics for ACI. Specifically, production of inflammatory cytokines, such as IL-6 and possibly other cytokines, leads to hepcidin-induced hypoferremia, resulting in iron sequestration within the reticuloendothelial system, thereby decreasing iron availability for erythropoiesis (Figure 5). The term ‘functional iron deficiency’ refers to insufficient iron availability at the site of erythroblast production, despite adequate body iron stores. It typically applies to the high hepcidin state in patients with renal insufficiency. However, broadly speaking, ACI, another high hepcidin condition, can also conceptually be referred to as functional iron deficiency. The teleological argument for the presence of anemia in conditions associated with inflammation is presumed to be related to iron sequestration, providing an evolutionary advantage in light of the iron dependence of pathogens and rapidly replicating cells. Thus, iron sequestration restricts iron availability and serves to limit growth of pathogens and malignant cells at the expense of hemoglobin synthesis. The specifics of iron regulation in infection with intracellular organisms (e.g. *Salmonella* and others) await additional clarification (Gehrer et al., 2023).

Furthermore, a newly emerging theme in inflammation-associated anemias is the contribution of alarmins, bioactive molecules released from damaged tissues or stressed cells. These factors may enter the circulation and engage receptors on target cells to promote anemia development. In models of sepsis, the release of the protein HMGB1 and of mitochondrial DNA act to suppress erythropoiesis and increase RBC turnover, respectively (Dulmovits et al., 2022; Lam et al., 2021). HMGB1 appears to directly block EPO-EPOR interaction, while mitochondrial DNA binds TLR9 on RBCs to enhance erythrophagocytosis and activate macrophage secretion of interferons. Notably, prior studies have implicated the alarmins S100A8 and S100A9 in the erythroid differentiation defect associated with a specific subclass of MDS, del(5q) (Schneider et al., 2016), raising the possibility that these factors may also contribute to inflammation-associated anemia. Finally, IL-33, recognized as both a cytokine and an alarmin, has been identified as a mediator of anemia in a murine model of inflammatory spondyloarthritis, acting directly on erythroid progenitors via its receptor, ST2 (Swann et al., 2020). While alarmin- or cytokine-induced anemia may be more directly causally linked to acute inflammation, similar mechanisms may potentially trigger chronically activated propagating loops, exemplified by the feed-forward relationship between erythrophagocytosis and macrophage activation (Lam et al., 2021). The role of iron restriction in alarmin-induced anemias remains to be established but is suggested by studies in which hepcidin neutralization or loss in a mouse model of acute inflammation ameliorates anemia (Sasu et al., 2010; Gardenghi et al., 2014).

IRIDA is a rare autosomal-recessive disorder caused by mutations in *TMPRSS6* (*transmembrane serine protease 6*) (Finberg et al., 2008). *TMPRSS6* is expressed primarily by the liver (Finberg et al., 2008; Hooper et al., 2003) and encodes matriptase-2, a member of a family of transmembrane serine proteases (Ramsay et al., 2008). Matriptase-2 acts as a negative regulator of BMP signaling for hepcidin production by cleaving the BMP co-receptor hemojuvelin from the cell membrane (Silvestri et al., 2008), and *TMPRSS6* mutations that impact the matriptase-2 catalytic domain result in impaired hemojuvelin cleavage. More recent evidence reveals that TMPRSS6 also targets other components of the BMP receptor complex by both proteolytic and nonproteolytic mechanisms (Enns et al., 2020; Krijt et al., 2021). As a consequence, patients with IRIDA exhibit hepcidin levels that are inappropriately elevated relative to their body iron stores.

Patients with IRIDA present with hypochromic, microcytic anemia (hemoglobin 6–9 g/dL), very low MCV (45–65 fL) and transferrin saturation (<5%), suppressed oral iron absorption, and abnormal iron utilization in response to parenteral iron. Surprisingly, infants with IRIDA demonstrate normal birth weights, normal growth and development, without cognitive concerns on long-term follow-up. Because these patients are generally healthy, anemia diagnosis is made via routine screening conducted in the first few years of life (Pearson and Lukens, 1999; Melis et al., 2008; Arsenault et al., 2016). Close to 50 different *TMPRSS6* mutations have been reported in IRIDA with most variants unique to individual families (Heeney and Finberg, 2014), and some evidence suggests linkage between common single-nucleotide polymorphisms in *TMPRSS6* and various hematological and iron-related laboratory parameters (Benyamin et al., 2009; Chambers et al., 2009; Ganesh et al., 2009; Soranzo et al., 2009). Taken together, although the pathophysiology underlying IRIDA has been elucidated, a robust understanding of the influence of TMPRSS6 mutations on hepcidin regulation and iron availability and the resultant compensatory mechanisms at various life stages that prevent a greater plethora of symptoms awaits discovery.

## What is ineffective erythropoiesis?

**Figure 6.**
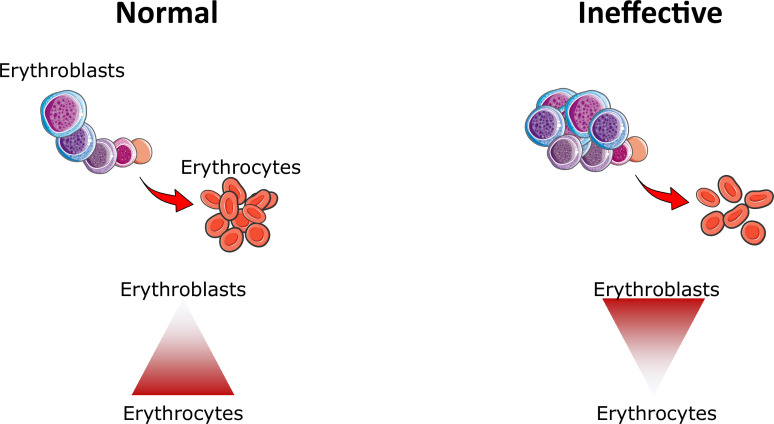
Ineffective erythropoiesis. Under normal conditions, small numbers of differentiating erythroblasts are needed to efficiently differentiate and enucleate to reticulocytes and ultimately mature red blood cells (erythrocytes). In conditions associated with ineffective erythropoiesis (e.g. β-thalassemia, myelodysplastic syndrome, and others), a block in erythroblast differentiation leads to the accumulation of immature erythroblasts, preventing efficient production of erythrocytes, resulting in anemia.

Ineffective erythropoiesis can be defined as the diminished production of enucleated RBCs despite an increase in the number of erythroid precursors (Figure 6). When the quantity and/or the ability of enucleated RBCs to transport oxygen declines below a certain level, patients require RBC transfusions for survival (Kattamis et al., 2022; Ginzburg and Rivella, 2011). Ineffective erythropoiesis has been a subject of intensive investigation in β-thalassemia, a disease in which ineffective erythropoiesis manifests with an expanded number of erythroid precursors and their reduced ability to differentiate, an increase in erythroblast death in these terminally differentiated cells, and reduced survival of enucleated RBCs (Ginzburg and Rivella, 2011). The proposed underlying mechanism leading to ineffective erythropoiesis in β-thalassemia is the relative excess of alpha-globin chains (EACs) (Kattamis et al., 2022; Ginzburg and Rivella, 2011). EACs alter erythropoiesis in at least two ways: in complex with free heme molecules, they form hemichromes, which are the main source of oxidative stress and cell death (Kattamis et al., 2022). In addition, EACs reduce the stability of GATA1, the main erythroid transcription factor in erythroblasts, interfering with their survival and maturation (Arlet et al., 2014). Furthermore, the abnormal and reduced number of RBCs in circulation lead to hypoxia-mediated EPO production, which in turn exacerbates further the expanded number of immature erythroid precursors (Libani et al., 2008).

Expansion in the number of erythroid precursors leads to increased ERFE production and consequent hepcidin suppression (Kautz et al., 2014). Hypoxia also plays a role by increasing expression of genes responsible for iron absorption in the duodenum (Anderson et al., 2013). Although in severely affected patients organ iron overload develops because of frequent RBC transfusions, in individuals with β-thalassemia who are not regularly transfused, the mechanisms responsible for increased iron absorption (i.e. insufficiently elevated hepcidin expression), along with chronic hemolysis, lead to progressive tissue iron deposition and toxicity, requiring even in these cases the use of iron chelators to prevent significant morbidity and mortality (Kattamis et al., 2022; Musallam et al., 2021).

Novel drugs and genetic approaches are now being translated to improve the quality of life in β-thalassemia (and other) patients or even cure them. Although a full description of these therapeutics is beyond the scope of the current review, these drugs can be broadly classified based on their mechanism of action as shown in Table 1. Among these, only gene therapy provides a curative approach. However, the risks remain high and alternative therapeutic options are welcome for those patients who are ineligible for cure. Drugs that act on iron metabolism (e.g. hepcidin-mimetics and transferrin) could also improve RBC quality and survival by limiting erythroid iron intake and hemichromes formation, as shown in mouse models of β-thalassemia (Li et al., 2010; Guo et al., 2013). However, even these drugs may fail to do so in β-thalassemia patients, their use could reduce iron absorption in combination with iron chelators if the patient is already iron overloaded. In addition, they could prevent iron from being accumulated in combination with drugs that improve RBC quality and production (e.g. luspatercept, mitapivat, and TFR2 inhibitors).

**Table 1.** Novel agents in development for β-thalassemia (and other) patients.

Mechanism of action	Agent name	Producer	Stage of development	Reference
Improve RBC quality and production	Luspatercept	BMS	FDA approved for TD β-thalassemia and MDS-RS	Cappellini et al., 2023
	Mitapivat	Agios	FDA approved for PKD; in phase II clinical trial for NTD α- and β-thalassemia patients	Kuo et al., 2022
	TFR2 inhibitors		Preclinical	Di Modica et al., 2022
Gene therapy to normalize the underlying genetic defect	Gene addition			Eckrich and Frangoul, 2023; Christakopoulos et al., 2023
	Gene editing			
Suppress erythropoiesis and prevent or reverse splenomegaly	JAK2 inhibitors	Novartis	Phase IIa (failed)	Taher et al., 2018
Alter iron import	Transferrin		Preclinical	Boshuizen et al., 2017
Limit iron absorption	Hepcidin agonist rusfertide	Protagonist	Currently in phase II and III clinical trials for PV patients	Handa et al., 2023
	Hepcidin inducer sapablursen	Ionis	Currently in phase II clinical trials for PV patients	Ganz et al., 2023
	Ferroportin inhibitor Vamifeport	Vifor	Currently in phase II clinical trials for SCD patients	Nyffenegger et al., 2022
	SLN124	Silence	Currently in phase I clinical trial for β-thalassemia and PV patients	
	ERFE inhibitors		Preclinical	Arezes et al., 2020

RBC, red blood cell; BMS, Bristol Myers Squibb; FDA, Food and Drug Administration; TD, transfusion dependent; MDS-RS, myelodysplastic syndrome with ringed sideroblasts; PKD, pyruvate kinase deficiency; NTD, non-transfusion dependent; TFR2, transferrin receptor 2; SCD, sickle cell disease; ERFE, erythroferrone.

Beyond the inherited forms of iron-loading anemias, that is, β-thalassemia, MDS is an acquired form of ineffective erythropoiesis associated with iron overload. MDS is a heterogeneous group of bone marrow stem cell disorders; several subtypes of MDS are characterized by ineffective erythropoiesis, leading to blood cytopenias and increased incidence of transformation to acute myeloid leukemia (Haferlach, 2019). The majority of MDS patients have a long median survival (e.g. 10 y), with 30–50% requiring only regular RBC transfusions to alleviate anemia and its associated symptoms (Dayyani et al., 2010; Kröger, 2019). RBC transfusions, however, are the main cause of progressive iron overload and consequent end-organ damage in transfusion-dependent MDS patients (Oliva et al., 2010). However, the risk–benefit ratio of treating iron overload in MDS patients remains controversial. Furthermore, RBC transfusion-dependence and iron overload correlate strongly with decreased survival in MDS patients (Malcovati et al., 2005; Malcovati et al., 2006; Garcia-Manero et al., 2008; Malcovati et al., 2011). In vitro experiments demonstrate that excess iron inhibits erythroid lineage differentiation in both murine and human hematopoietic progenitors, exhibiting dysplastic changes with increased intracellular reactive oxygen species (ROS), decreased expression of anti-apoptotic genes, and DNA damage, triggering apoptosis, and worsening disease in MDS (Pan et al., 1999; Camaschella et al., 2007; Fibach and Rachmilewitz, 2012; Taoka et al., 2012; Hartmann et al., 2013).

The TELESTO trial was designed and executed to test whether iron chelation provides clinical benefit in iron-overloaded lower risk MDS patients (Angelucci et al., 2020). The results demonstrate prolonged event-free survival in deferasirox-treated MDS patients without clear improvement in hemoglobin, reduction of RBC transfusion burden, or effect on overall survival. We hypothesize, based on evidence from mutant monoferric mice (see above), that the lack of effect of deferasirox on erythropoiesis is a consequence of the specific iron chelator selected. Deferasirox and most other commercially available iron chelators have a higher iron binding affinity relative to transferrin (Sohn et al., 2008); only deferiprone iron-chelating effect enables iron transfer from parenchymal cells to increased transferrin saturation (Schmidt et al., 2015; Casu et al., 2016a), potentially modulating monoferric transferrin concentrations. Furthermore, increasing transferrin saturation itself results in increased hepcidin expression in the liver (Schmidt et al., 2015; Casu et al., 2016b), in turn preventing further iron absorption and recycling, trapping iron within macrophages (Ganz, 2005), and decreasing iron availability for erythropoiesis to potentially ameliorate ineffective erythropoiesis in MDS. A direct beneficial effect of DFP on erythropoiesis in MDS has yet to be demonstrated. Recent data demonstrates partial reversal of ineffective erythropoiesis in addition to iron overload, normalizing erythroblast iron trafficking and restoring EPO responsiveness in deferiprone-treated mouse model of MDS, NUP98-HOXD13 transgenic mice (An et al., 2022).

In addition, a specific subtype of low-risk MDS, namely MDS with ringed sideroblasts, occurs in 20% of all MDS patients. Splicing factor 3 B subunit 1 (*SF3B1*) mutations in hematopoietic stem and progenitor cells is a hallmark of this disease, inducing aberrant splicing of genes involved in heme biosynthesis and mitochondrial iron transport, leading to the abnormal deposition of iron in erythroblasts, and resulting in dysfunctional hemoglobin synthesis and formation of ringed sideroblasts (Visconte et al., 2015; Dolatshad et al., 2015; Dolatshad et al., 2016; Shiozawa et al., 2018; Clough et al., 2022). Although preclinical data in mouse models predicted a therapeutic effect of splicing inhibition, a recent phase I clinical trial did not yield significant clinical improvement (Lee et al., 2016; Steensma et al., 2021). Growing evidence suggests that the heme-regulated EIF2AK1 kinase pathway affects erythropoiesis in health and disease. For example, EIF2AK1 effector DDIT3 is overexpressed in MDS hematopoietic stem and progenitor cells (Berastegui et al., 2021), DDIT3 overexpression delays erythroid differentiation in CD34-positive cells from MDS patients, and EIF2AK1 inhibition increases expression of both mitochondrial heme biosynthesis enzymes and iron transporters, reversing *SF3B1* mutation-induced arrest of erythroid differentiation in vitro (Adema et al., 2022).

Lastly, novel therapy has recently been US Food and Drug Administration (FDA) and European Medicines Agency (EMA)-approved for very low- to intermediate-risk MDS with ringed sideroblasts. Luspatercept is a modified activin receptor IIB ligand trap, a member of the transforming growth factor-β (TGF-β) superfamily. While this agent was studied in multiple different MDS subgroups of patients in a phase II trial, its efficacy was most robust in patients with MDS with ringed sideroblasts (Platzbecker et al., 2017) and led to the phase III MEDALIST trial, a double-blind, placebo-controlled, multicenter study in transfusion requiring patients with MDS with ringed sideroblasts (Fenaux et al., 2018). In the MEDALIST trial, luspatercept led to transfusion independence for >8 wk in 45% of enrolled subjects and median duration of response was 6 mo (Farrukh et al., 2022). To consider how MDS with ringed sideroblasts is unique, prior evidence demonstrates that these patients exhibit iron overload prior to the initiation of RBC transfusion (Gattermann, 2005) likely as a consequence of especially suppressed hepcidin relative to other MDS subtypes (Gu et al., 2013; Santini et al., 2011) and *HFE* gene polymorphisms that predispose to iron overload are detected in up to 21% of MDS with ringed sideroblasts, significantly higher than in other MDS subtypes (Nearman et al., 2007; Valent et al., 2008). A more complete understanding of how the formation of ringed sideroblasts in MDS contributes to worsening ineffective erythropoiesis is currently lacking. Taken together, these recent findings provide supporting evidence for dysregulated iron trafficking in the pathophysiology of ineffective erythropoiesis in MDS.

## Iron metabolism dysregulation in polycythemia vera

Polycythemia vera (PV), one of the chronic myeloproliferative neoplasms, is a clonal hematopoietic stem cell disorder driven by EPO hypersensitive signaling via the JAK2-STAT5 pathway, resulting in excess proliferation of erythroid precursors (Rampal et al., 2014; Levine et al., 2005; Baxter et al., 2005; Kralovics et al., 2005; James et al., 2005; Lu et al., 2008; Akada et al., 2010). The vast majority of PV patients are the result of acquired *JAK2* mutations in their stem cells, namely *JAK2*V617F on exon 14 (95% of PV patients) or mutations in exon 12 of the *JAK2* gene (2–3% of PV patients) (Pardanani et al., 2007; Scott et al., 2007). PV patients are frequently iron deficient at the time of diagnosis (Gianelli et al., 2008; Thiele et al., 2001; Kwapisz et al., 2009), and this is further exacerbated by therapeutic phlebotomies administered with the goal of maintaining hematocrit below 45% to decrease thrombotic risk (Marchioli et al., 2013). Repeated phlebotomies may in part dampen erythropoiesis by inducing iron deficiency but also potentially contribute to PV-associated systemic symptoms due to the depletion of iron stores in non-hematopoietic tissues (Pratt and Khan, 2016). Recent analysis of PV patients treated with ruxolitinib, a JAK1/2 inhibitor, corroborates the role of iron deficiency in the manifestations of this disease as symptom improvement with ruxolitinib is at least partly attributable to reversal of systemic iron deficiency (Verstovsek et al., 2017).

We previously demonstrate that PV, compared to secondary forms of erythrocytosis, is associated with relative suppression of hepcidin, potentially due to more expanded erythropoiesis and iron depletion (Ginzburg et al., 2018). In addition, PV patients experience erythrocytosis despite a more profound iron deficiency relative to healthy blood donors (Feola et al., 2019; Stetka et al., 2023). This is evidenced by significantly lower MCVs, serum iron and ferritin concentrations, and transferrin saturation; this systemic iron deficiency in PV patients does not resolve despite elevated ERFE with consequent hepcidin suppression. Hepcidin suppression would be expected to result in enhanced intestinal iron absorption and mobilization of intracellular recycled and stored iron, leading to cellular iron efflux, more circulating iron, and recovery from systemic iron deficiency. However, recovery from iron deficiency does not occur in PV, where a low hepcidin state is insufficient to replenish iron stores, implying dysregulated iron homeostasis.

We hypothesize that relative hepcidin suppression without recovery from iron deficiency in PV may result from the combined effects of concurrent inflammation, insufficiently elevated ERFE with insufficiently suppressed hepcidin, and/or aberrant hypoxia signaling in the intestine preventing recovery from iron deficiency (Nemeth and Ganz, 2014; Frise et al., 2016; Shah et al., 2009). A recent report suggests that ERFE exerts a relatively diminished effect on hepcidin regulation relative to that of inflammation in PV patients (Bennett et al., 2023). In this study, *Erfe* deletion in *Jak2*^V617F^ mice did not alter hepcidin levels or disease severity, resulting in stably elevated hematocrits and RBC counts. Using a human hepatocyte cell line, HepG2 cells, the authors further explored the hypothesis that PV-associated inflammation leads to hepcidin upregulation, demonstrating that the increased hepcidin expression was induced by plasma from PV patients but not plasma from normal controls and was normalized by blocking IL-6 binding to its receptor (Bennett et al., 2023). These findings suggest that inflammatory cytokines in PV may be crucial to disordered iron utilization. In addition, persistent erythropoiesis despite iron deficiency in PV may also occur as a consequence of aberrant erythropoiesis that is insensitive to iron deficiency, preventing physiological mechanisms that normally coordinate iron supply with erythropoietic output (Khalil et al., 2017; Khalil et al., 2018; Feola et al., 2019). An excellent review on dysregulated iron metabolism in PV was recently published (Ginzburg et al., 2018).

To elucidate briefly, suboptimally suppressed hepcidin prevents recovery from iron deficiency, enables absorption of iron to maintain pathologically enhanced erythropoiesis, and provides a rationale for maximizing this finding for therapeutic purposes in PV. Said another way, although we do not yet understand the pathophysiological mechanism that enables persistent erythropoiesis in PV despite iron deficiency, we anticipate that using hepcidin mimetics to further suppress iron absorption and recycling may prevent erythropoiesis in PV, redistributing iron to non-hematopoietic cells and possibly reversing iron deficiency associated symptoms in PV patients.

**Figure 7.**
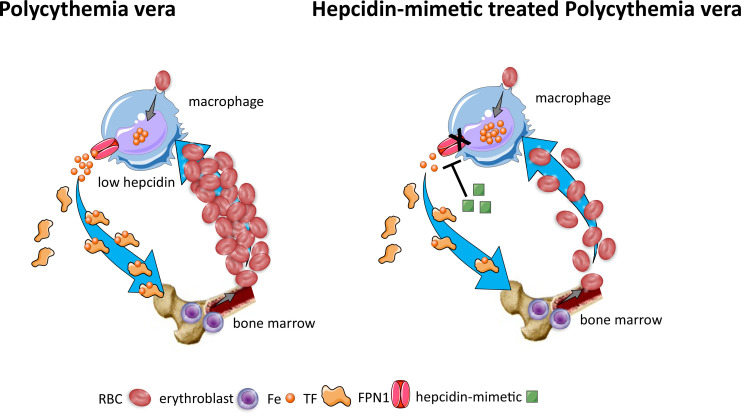
Effects of hepcidin-mimetic on erythropoiesis in polycythemia vera. Similar to normal erythropoiesis, in polycythemia vera, iron recycling from multiple sources within macrophages leads to export of iron via ferroportin back into the circulation where it is loaded onto transferrin and delivered to cells with iron requirements (e.g. for hemoglobin synthesis in erythroblasts during erythropoiesis in the bone marrow). Unlike normal erythropoiesis, erythropoiesis proceeds despite iron deficiency and hepcidin remains low, enabling continued iron release into the circulation to support continued erythropoiesis. Increased hepcidin leads to binding to and occlusion of the ferroportin channel, preventing iron egress from cells involved in iron recycling (e.g. splenic red pulp macrophages), leading to the accumulation of iron within cellular ferritin core, and causing decreased iron-bound transferrin (low transferrin saturation). This decreased availability of iron for erythropoiesis results in reduction of erythrocytosis in polycythemia Vera. RBC, red blood cell; Fe, iron; TF, transferrin; FPN, ferroportin 1.

Lastly, significant advances in the translation of hepcidin mimetics in PV are worth noting. Our understanding about hepcidin’s mechanism of action predicts that hepcidin elevation would be expected to sequester recycled and stored iron and prevent iron absorption, resulting in reduced iron availability for erythropoiesis and replenishing iron stores within liver and splenic macrophages, thus aiding in recovery from systemic iron deficiency (Casu et al., 2018, Aschemeyer et al., 2018; Ginzburg et al., 2018; Ginzburg, 2019; Figure 7). Preclinical studies demonstrated proof of principle for this approach using minihepcidins, engineered peptides with the necessary functional ferroportin binding domain (Preza et al., 2011), resulting in a significant dose-dependent decrease in RBC count, hematocrit, and splenomegaly in *Jak2*^V617F^ mice, a well-established mouse model of PV (Casu et al., 2016b). In addition, minihepcidin results in increased iron in the splenic red pulp of *Jak2*^V617F^ mice, consistent with sequestration of recycled iron. More recently, another hepcidin mimetic agent—antisense oligonucleotide targeting TMPRSS6, leading to the downregulation of TMPRSS6 gene product that prevents the degradation of HJV, yielding an increase in endogenous hepcidin expression in the liver—used in *Jak2*^V617F^ mice also resulted in decreased RBC counts and hematocrit levels as well as suppression of bone marrow erythroblast numbers (Casu et al., 2021). Similar findings were also recently demonstrated using a parenteral synthetic hepcidin (Taranath et al., 2021) and an orally bioavailable ferroportin inhibitor (Stetka et al., 2023).

Most recently, preliminary results from phase II clinical trials evaluating the safety and efficacy of hepcidin mimetic rusfertide (PTG-300) in phlebotomy-requiring PV patients demonstrate a virtual elimination of phlebotomy requirements, control of RBC count, increase in systemic iron stores, and a potential decrease in systemic symptoms (Hoffman, 2021; Hoffman et al., 2022; Ginzburg et al., 2021). A dramatic reduction in phlebotomy requirements, with 84% of PV subjects achieving phlebotomy-independence, was observed in the first 28 wk of treatment, and hematocrit control was sustained for up to 2 y on study drug. Several other hepcidin-inducing agents are either enrolling PV patients to a phase II clinical trial (NCT05143957) or in planning stages, and the global, multicenter, randomized, placebo-controlled phase III trial (NCT05210790) is currently underway (Verstovsek et al., 2021) to further clarify the potential role of rusfertide in the management of patients with PV.

## What role do macrophages play in supporting normal and disordered erythropoiesis?

Erythropoiesis occurs at the erythroblastic island (EBI) that is composed of a central macrophage surrounded by developing erythroid cells (Bessis, 1958) and granulocyte progenitors (Romano et al., 2022). The functional role of EBI was first suggested by Mohandas and colleague, who showed that in hyper-transfused rats, the numbers of EBI in the bone marrow were significantly decreased (Mohandas and Prenant, 1978). The importance of the central macrophage in supporting normal erythropoiesis was further supported by the abnormal macrophage differentiation in EMP-null (Wei et al., 2019; Soni et al., 2006), KLF1-null (Mukherjee et al., 2021; Porcu et al., 2011), and other mouse models (Chow et al., 2013; Sadahira et al., 1995; Kawane et al., 2001; Mankelow et al., 2004), leading to significantly impaired erythropoiesis and anemia. Furthermore, the depletion of macrophages with either clodronate liposomes or CD169-diptheria toxin leading to impaired erythropoiesis provide direct evidence that macrophages play critical roles in supporting erythropoiesis in vivo, particularly during stress erythropoiesis (Chow et al., 2013; Ramos et al., 2013). In vitro studies showed that macrophages promoted erythroblast proliferation/survival (Rhodes et al., 2008; Lopez-Yrigoyen et al., 2019; Perron-Deshaies et al., 2020). It has been reported that fetal liver macrophages can efficiently engulf extruded nuclei in a phosphotidylserine-dependent manner (Yoshida et al., 2005) and that failing to degrade the engulfed DNA by macrophages due to lack of DNAase II led to severe anemia and embryonic death (Sadahira et al., 1995). Notably, in both *Jak2*^V617F^ (PV mouse model) and *Hbb*^3/+^ (transfusion independent β-thalassemia mouse model) mice, macrophage depletion normalized erythropoiesis (Ramos et al., 2013). Together, these findings indicate that macrophages play important roles in supporting normal erythropoiesis and can be targeted to at least partly ameliorate the disordered erythropoiesis in PV and β-thalassemia. However, due to the inability to identify and isolate EBI macrophages for cellular and molecular studies, the mechanisms by which EBI macrophages support normal erythropoiesis or contribute to disordered erythropoiesis have not been fully interrogated. We recently discovered that EBI macrophages are characterized by the expression of EPOR (Li et al., 2019; Zhang et al., 2021) and that EPO enhanced the ability of macrophages to form EBIs with erythroblasts both in vitro and in vivo (Li et al., 2019). Supporting the functional role of EPO/EPOR in EBI macrophages, others also documented that Epo/EpoR signaling in macrophages is required for stress erythropoiesis in the spleen (Chen et al., 2020). In addition, fine characterization of EBIs indicates neutrophil precursors specifically associated with BM EBI macrophages, suggesting that erythro-(myelo)-blastic islands are a site for terminal granulopoiesis and erythropoiesis (Romano et al., 2022). Finally, the relative proportion of granulocytes within EBIs increases during inflammatory conditions and decreases during stress erythropoiesis, suggestive of a functional plasticity of the central macrophage within the EBIs (Romano et al., 2022).

To develop a comprehensive characterization of the EBI macrophages at the molecular level and gain insights into the mechanisms by which they support erythropoiesis, we performed RNA-seq analyses on the sorted bone marrow F4/80^+^EpoR^+^ and F4/80^+^EpoR^-^ macrophages (Li et al., 2019). Bioinformatics analyses revealed that the expression levels of *Vcam1* and *CD169* known to be involved in macrophage–erythroblast interaction (Chow et al., 2013; Sadahira et al., 1995) were significantly higher in F480^+^EpoR^+^ than in F480^+^EpoR^-^ macrophages. Similarly, the expression levels of *Mertk* required for pyrenocyte engulfment (Toda et al., 2014), and DNase2α (*DNAse2*) critical for DNA degradation of the engulfed nuclei (Kawane et al., 2001), were also significantly higher in the F480^+^EpoR^+^ macrophages. Intriguingly, key molecules involved in iron recycling such as phosphotidylserine receptor Tim4, heme oxygenase-1, iron exporter ferroportin, and iron transporter transferrin are also abundantly expressed in bone marrow F4/80^+^EpoR^+^ macrophages. In addition, insulin growth factor 1, one of the known erythropoiesis-promoting cytokines, is expressed in EBI macrophages but not non-EBI macrophages. These findings provide support for the long-standing expectation that EBI macrophages are unique in providing essential elements to enable differentiation of the surrounding erythroblasts. Further evidence is forthcoming regarding the mechanistic nature of the support EBI macrophages provide to differentiating erythroblasts.

## Tools and analytic endpoints for studying erythropoiesis and iron metabolism

To study erythropoiesis, it is important to identify and isolate erythroid lineage cells at distinct stages of differentiation. During the past decade, considerable progress has been made, and methods for analyzing and isolating murine and human erythroblasts at distinct developmental stages have been developed. Here, we summarize these methods.

### Isolation of murine erythroid progenitors

Traditionally, the erythroid progenitors BFU-E and CFU-E have been functionally defined by their ability to form erythroid colonies of distinct kinetics and morphology (Iscove and Sieber, 1975; Gregory and Eaves, 1977). It should be pointed out that the erythroid colonies contain terminally differentiated erythroid cells and not the BFU-E and CFU-E cells themselves. With the development of flow technology for analysis and sorting of cells using lineage-specific surface markers, a flow cytometry-based method was developed to isolate erythroid progenitors from mouse fetal liver (Flygare et al., 2011). Briefly, the fetal liver lineage^+^ cells were depleted by antibodies against murine Ter119, B220, CD3, Gr-1, CD41, Sca-1, CD34, Mac-1, and CD16/CD32. The resulting lineage^-^ cells were stained with CD117 (c-Kit) and CD71. Within the Kit^+^ fraction, the level of CD71 expression was used to separate BFU-E (CD71^10%low^) and CFU-E (CD71^20%high^) with more than 90% purity (Flygare et al., 2011). A similar strategy can be used to isolate murine bone marrow erythroid progenitors, but unlike fetal liver BFU-E, the bone marrow BFU-E cells are Kit^+^CD71^-^ (Zhang et al., 2021).

### Isolation of human erythroid progenitors

To identify the surface markers for human BFU-E and CFU-E, we systematically examined the expression of surface markers CD34, IL-3R, CD36, CD71, CD45, and GPA during human early stage erythropoiesis in vitro. Based on the expression profiles of these surface markers and the related colony-forming ability, the surface marker profiles for human BFU-E and CFU-E are CD45^+^GPA^-^IL-3R^-^CD34^+^CD36^-^CD71^low^ and CD45^+^GPA^-^IL-3R^-^CD34^-^CD36^+^CD71^high^, respectively (Li et al., 2014). Importantly, this method can be used to isolate primary BFU-E and CFU-E cells from human bone marrow, umbilical cord blood, and peripheral blood (Li et al., 2014). A recent study documented that early human erythroid progenitors can be further subdivided into four subpopulations as they lose CD34 staining and acquire CD105 during progression from BFU-E to immature CFU-E and sequentially mature CFU-Es (Yan et al., 2021).

### Isolation and quantification of murine erythroblasts terminal differentiation

To identify surface markers for isolating murine erythroblasts, we examined the changes in RBC membrane proteins during murine terminal erythroid differentiation and found that the expression of CD44 dramatically decreased during erythroid differentiation, with more than a 30-fold decrease from Pro to Ortho erythroblasts. Use of CD44 in conjunction with erythroid lineage marker TER119 and forward scatter (cell size) enabled stage-specific purification of murine erythroblasts with more than 90% purity (Chen et al., 2009). Under physiological conditions, murine Pro undergo three rounds of mitosis to sequentially generate Baso, Poly, and Ortho erythroblasts. It is therefore expected that during normal terminal erythroid differentiation the ratio of Pro:Baso:Poly:Ortho should follow a 1:2:4:8 pattern. We further improved this method, enabling quantification of this process in vivo, and identified stage-specific alterations during terminal erythroid differentiation of β-thalassemia mouse bone marrow (Liu et al., 2013).

### Isolation and quantification of human erythroblasts during terminal differentiation

To identify surface markers for staging human erythroblasts, we examined changes in surface markers during human terminal erythroid differentiation in vitro. Notably, different from mouse, CD44 demonstrates no significant changes during terminal erythropoiesis. Interestingly, while cell surface band 3 progressively increases, α4 integrin decreases. The use of band 3 and α4 integrin in conjunction with the human erythroid lineage marker glycophorin A enabled separation of highly purified populations of erythroblasts at distinct stages in culture, designated as Pro (α4 integrin^hi^band3^neg^), early Baso (α4 integrin^hi^band3^low^), late Baso (α4 integrin^hi^band 3^med^), Poly (α4 integrin^med^band3^med^), and Ortho (α4 integrin^low^band 3^hi^) erythroblasts (Hu et al., 2013). Importantly, the surface markers identified using the in vitro erythroid culture system can be used to separate erythroblasts at distinct developmental stages from primary human bone marrow cells. Furthermore, the ratio of erythroblasts at successive stage in human bone marrow followed the predicted 1:2:4:8:16 pattern. Analyses of bone marrow from patients with MDS and sickle cell disease revealed the expected alteration in terminal erythroid differentiation profiles (Hu et al., 2013; Ali et al., 2018; El Hoss et al., 2021). These methods offer novel strategies for quantitative assessment of erythroid differentiation in mouse disease models and human erythroid disorders.

### Assessment of enucleation by flow cytometry

Enucleation is the process during which the condensed nucleus is extruded from the erythroblast to yield the reticulocyte and the ‘pyrenocyte.’ Discrimination of nucleated erythroblasts, reticulocytes, and extruded nuclei by flow cytometry is based on DNA staining, surface expression of erythrocyte-specific markers, or forward scatter. The enucleation of murine erythroblasts is assessed by surface expression of the murine erythrocyte marker TER119 and DNA staining (Ji et al., 2008; Zhang et al., 2003). Three discrete populations that represent nucleated erythroblasts, reticulocytes, and extruded nuclei are defined as Hoechst^med^TER119^high^, Hoechst^low^TER119^high^, and Hoechst^high^TER119^med^, respectively (Ji et al., 2008; Zhang et al., 2003). Another nuclei acid staining dye, SYTO16, is used for the assessment of human enucleation in combination with forward scatter. For human cells, the three populations that represent nucleated erythroblasts (high forward scatter SYTO16^+^), reticulocyte (high forward scatter SYTO16^-^), and extruded nuclei (low forward scatter SYTO16^+^) are thus identified (Yoshida et al., 2005).

### Models for iron-restricted anemias

Cell culture and in vivo techniques have been developed for analysis of the effects of iron restriction on erythropoiesis. In in vitro cell culture, human or murine hematopoietic stem and progenitors are subjected to a two-stage system using defined, serum-free conditions. Progenitors successfully studied have included human CD34^+^ peripheral blood-mobilized progenitors and murine Lin^-^Kit^+^ splenic stress progenitors (Bullock et al., 2010; Khalil et al., 2018). In the initial phase of culture, progenitors undergo expansion for ~2 d in the presence of early-acting cytokines, SCF, FLT3-ligand, TPO, and IL-3. The cells are then shifted into erythroid medium containing SCF and EPO. By adding different proportions of apo- and holo-transferrin, the transferrin saturation (%TSAT) can be adjusted to create iron-replete or iron-restricted conditions. Initial studies with human progenitors identified a TSAT level of 15% as showing a selective inhibition of erythropoiesis while not affecting granulopoiesis or megakaryopoiesis (Bullock et al., 2010). In subsequent studies with human stem and progenitor cultures, either TNFα or IFNγ when combined with a TSAT of 15% (instead of 100%) resulted in a synergistic suppression of erythropoiesis, suggesting a means for modeling ACI in vitro (Richardson et al., 2013). The most straightforward in vivo model for iron-restricted anemia consists of placing mice on low iron diet using customized mouse chow (Envigo Teklad 2.5–4 ppm iron). Important considerations in this model are to use male weanlings, enhance susceptibility to the development of iron deficiency, and use control customized iron-replete chow (containing ~35–50 ppm) that is matched in composition to the low iron chow. Robust in vivo models for iron-restricted anemia in the setting of chronic inflammation have included rat adjuvant arthritis, caused by injection of the streptococcal cell wall peptidoglycan-polysaccharide and murine chronic inflammation induced by weekly injections of low dose killed *Brucella abortus* combined with customized iron-replete chow (Envigo Teklad 35–50 ppm) (Richardson et al., 2013; Guo et al., 2019; Goldfarb et al., 2021). Evidence supporting a role for iron restriction in these rodent ACI models consisted of the amelioration of the anemia with isocitrate injections (Richardson et al., 2013; Goldfarb et al., 2021). Finally, as discussed in respective section above, several mouse models that recapitulate diseases such as β-thalassemia, MDS, and PV are commercially available.

## Perspectives and future directions (all)

Taken together, a great deal is currently known about the physiology of erythropoiesis and iron metabolism as well as the pathophysiology of diseases in which these biological systems are dysregulated. Despite this, significant unknowns remain, both regarding the mechanisms of normal function and their disordered regulation in disease; we thus include here an incomplete list to guide the next generation of targeted inquiry along these lines:

- A mechanistic molecular understanding of ineffective erythropoiesis and a more complete delineation of how EPO responsiveness is modulated in both physiological and pathological conditions remains elusive. This direction of investigation could yield novel therapeutic development for a variety of diseases associated with anemia, for example, anemia of chronic inflammation and in renal failure.
- The role of and purpose for iron and heme export from erythroblasts (via ferroportin and FLVCR, respectively) is counterintuitive and incompletely understood.
- Whether and how iron-specific proteins (Tfr1, Tfr2, transferrin, etc.) in non-hepatocyte cells regulate the crosstalk between iron metabolism, immunity, and erythropoiesis is not well understood. Such exploration may yield novel therapeutic targets for a variety of disorders.
- Whether and how EPOR expression outside of erythroid lineage cells regulate the crosstalk between iron metabolism and erythropoiesis remains to be more completely elucidated.
- How iron deficiency anemia exerts and influences platelet production remains largely unknown despite the long-standing clinical recognition of thrombocytosis as a common co-occurrence. Such mechanisms could potentially be exploited in designing novel treatments for thrombocytopenia.
- Genetic factors most likely contribute to heterogeneity in the human response to iron deficiency. Why do individuals with the same degree of iron deficiency show differences in the extent of anemia and of thrombocytosis? Understanding such factors may enable a more personalized approach toward iron-related therapies.
- A novel mechanism for cell death, ferroptosis, was recently discovered, an iron-mediated mechanism leading to lipid peroxidation and cell death that is being targeted as a therapeutic approach in various cancers. Understanding the role of ferroptosis as it pertains to iron trafficking in erythroblasts may shed light on iron-loading anemias associated with ineffective erythropoiesis (i.e. β-thalassemia and MDS).
- Given the critical role of EBI macrophages in supporting erythropoiesis, understanding whether and how EPO/EPOR signaling in these cells further enables coordination of erythropoiesis and enucleation in normal and disordered erythropoiesis is important.
